# Ruxolitinib improves symptoms and quality of life in a patient with systemic mastocytosis

**DOI:** 10.1186/s40364-016-0056-5

**Published:** 2016-02-05

**Authors:** Abdulraheem Yacoub, Lindsey Prochaska

**Affiliations:** Division of Hematology and Oncology, Univerity of Kansas Medical Center, 2330 Shawnee Mission Parkway, 66205 Westwood, Kansas USA

**Keywords:** Systemic mastocytosis, Mastocytosis, Ruxolitinib, Quality of life

## Abstract

**Background:**

Systemic mastocytosis is a clonal myeloproliferative neoplasm associated with constitutional symptoms from mast cell mediated chemical and cytokine release. According to the literature, Ruxolitinib, a JAK1/JAK2 inhibitor, has been shown to reduce symptoms related to proinflammatory cytokine release in other myeloproliferative neoplasms.

**Case presentation:**

Here we present a case using Ruxolitinib for disabling constitutional symptoms despite complete bone marrow response in a patient with aggressive systemic mastocytosis. Assessment tools used to monitor symptoms in previously published Ruxolitinib trials were adopted to track symptom improvement and quality of life.

**Conclusions:**

Ruxolitinib significantly improved symptoms and quality of life in our patient with systemic mastocytosis.

## Background

Systemic mastocytosis (SM) is a very rare clonal myeloproliferative neoplasm (MPN) [1]. The hallmarks of disease are mast cell (MC) degranulation and organ infiltration by multifocal clusters of abnormal mast cells, including bone marrow. Aggressive systemic mastocytosis (ASM) presents with symptoms related to organ infiltration by mast cells, including dermatologic, hematologic, gastrointestinal, and skeletal manifestations [2]. Mast cell degranulation leads to various cytokine release, producing pruritus, flushing, dyspepsia, hypotension, and even shock [2]. The workup of SM includes testing for mutations that play a role in pathogenesis and treatment implications. Many of the molecular defects associated with SM involve activating mutations in the gene encoding the c-kit receptor, the most common of which is KIT mutation D816V [3, 4]. D816V, N8221 mis-sense mutation, and Val559lle juxtamembrane-type mutation are all KIT mutations that render imatinib resistance in mast cells [5]. There are some mutations not associated with the activation loop of KIT, such as K5091 mutation and Phe522Cys KIT mutation, in which imatinib can be effective [5]. Cytoreductive agents have been used in attempts to control ASM, but treatment remains challenging and mostly palliative. Clinical trials are investigating treatment with KIT D816V inhibitors in relapsed or refractory disease. Cytokine release symptoms are typically treated with histamine receptor antagonists and glucocorticoids, but can remain debilitating despite systemic disease control [6].

Ruxolitinib, a potent inhibitor of JAK1 and JAK2, has been shown in the literature to reduce symptoms related to proinflammatory cytokine release in hematologic diseases. The COMFORT-I and COMFORT-II trials using Ruxolitinib showed a significant decrease in disease related symptoms in patients with myelofibrosis [7, 8]. The RESPONSE trial showed similar results with significant symptom improvement in patients with polycythemia vera using Ruxolitinib [9]. Here we present a case using Ruxolitinib for disabling constitutional symptoms despite complete bone marrow response in a patient with aggressive SM. Myeloproliferative Neoplasm Symptom Assessment Forms (MPN-SAF), the European Organisation for Research and Treatment of Cancer (EORTC) Quality of Life Questionnaire–Core 30 Version 3.0 (QLQ-C30), Brief Fatigue Inventory (BFI), and Patient Global Impression of Change (PGIC) were used to assess symptom response [7–10].

Permission to use the BFI was granted by The University of Texas M. D. Anderson Cancer Center. EORTC Quality of Life Group granted permission to use the EORTC QLQ. HIPPA approval was obtained and approved by KUMC IRB.

## Case presentation

A 30-year-old woman was diagnosed with aggressive systemic mastocytosis at age 9 after battling with cutaneous and gastrointestinal symptoms for 4 years. At age 24, she suffered significant progression of her symptoms with disabling fatigue, flushing, and chronic bone pain requiring high doses of narcotic analgesia. Bone marrow biopsy at that time showed 50 % involvement with mast cells and her tryptase level was 101 ng/mL. KIT mutation analysis showed a rare K509I mutation that is sensitive to imatinib [11]. She was started on imatinib 100 mg daily and achieved complete bone marrow response and normalization of tryptase level. Despite disease control, patient continued to suffer from significant constitutional symptoms.

Subsequent bone marrow biopsy continued to showed normocellular marrow with no abnormal mast cells. There was an increase in reticulin fibrosis that was not previously reported. Jak-2 mutation was negative and cytogenetics were normal.

Imatinib was continued, but Ruxolitinib was started in attempt to control symptoms. Ruxolitinib was initiated at 5 mg twice daily and titrated up every 4 weeks over a 24-week period based on symptoms response and tolerance. Patient was monitored closely for toxicity or adverse effects from the addition of Ruxolitinib. Weight, EKG, and labs including CBC, CMP, amylase, lipase, and tryptase were collected at baseline and monitored weekly.

Labs and clinical data were recorded with each Ruxolitinib dose adjustment and results are depicted in Table 1. Only mild anemia was seen with increasing doses of Ruxolitinib that reversed once dose was decreased. No additional cytopenias were noted. Liver function, amylase, and lipase remained at baseline. Tryptase remained stable. No EKG changes were seen. Weight gain was witnessed over the 24-week period.

**Table 1:** Patient characteristics

	Baseline	Week 4	Week 8	Week 12	Week 16	Week 20	Week 24
Hemoglobin	12	11.7	11.3	10.5	10.9	11.4	11.6
Platelet count	188	246	277	297	314	296	250
WBC	6.3	6.8	6.9	7.3	7.4	8.0	8.3
Creatinine	0.85	0.88	0.89	0.92	1.02	1.16	1.03
ALT/AST	Normal	Normal	Normal	Normal	Normal	Normal	Normal
Amylase/Lipase	Normal	Normal	Normal	Normal	Normal	Normal	Normal
EKG	Normal	Normal	Normal	Normal	Normal	Normal	Normal
Weight (Kg)	77.9	78.4	80.5	83.7	85.2	85.6	84.6
Tryptase	10.2	NA	10.8	9.6	NA	9.5	9.1
Ruxolitinib dose	5 mg BID	10 mg BID	20 mg BID	15 mg BID	10 mg BID	10 mg BID	5 mg BID

**Fig. 1:**
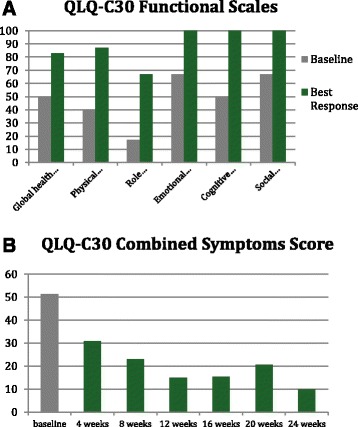
QLQ-C30 Functional scales (**a**) and combined symptoms score (**b**)

**Fig. 2:**
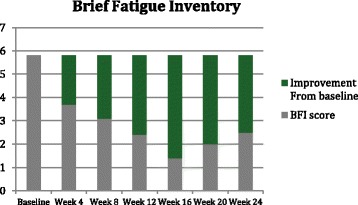
Brief fatigue inventory

**Fig. 3:**
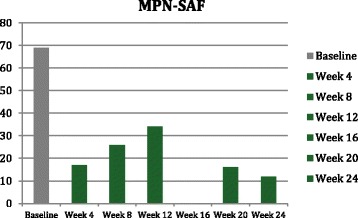
MPN-SAF

**Fig. 4:**
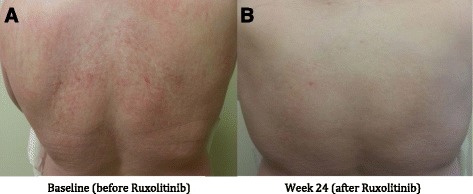
Skin rash prior to therapy with Ruxolitinib (**a**) and 24 weeks post therapy (**b**)

Symptoms and quality of life were assessed every 4 weeks by EORTC QLQ-C30 (Fig. 1a and b), BFI (Fig. 2), MPN-SAF (Fig. 3), and PGIC. Patient reported significant improvement in quality of life due to symptom reduction. Narcotic requirement was reduced from Methadone 30 mg daily to 15 mg daily. Flushing resolved and cutaneous manifestations markedly improved (Fig. 4a and b).

## Conclusions

Systemic mastocytosis is associated with constitutional symptoms from mast cell mediated cytokine release that occurs chronically and episodically. Ruxolitinib is a Jak1/Jak2 inhibitor that blocks signal transduction for many cytokine receptors, leading to its effectiveness in patients with myeloproliferative neoplasms (MPNs).

In the COMFORT-I trial, patients with myelofibrosis were treated with Ruxolitinib and had significant improvement in the total symptom score using the modified Myelofibrosis Symptom Assessment Form (MFSAF). MFSAF scores for abdominal pain, pain under the ribs on the left side, and early satiety, which were all improved. The PGIC and patient-reported outcomes also illustrated symptom improvement. The COMFORT-2 trial also evaluated the use of Ruxolitinib in myelofibrosis patients. Symptoms and quality of life in this trial were assessed by the EORTC QLQ-C30 and Functional Assessment of Cancer Therapy-Lymphoma scale (FACT-Lym). This study showed improvements in quality of life and functioning in the patients receiving Ruxolitinib. Significant reductions were seen in fatigue, weight loss, dyspnea, insomnia, and pain [7, 8]. The RESPONSE trial randomized patients with polycythemia vera who were refractory or intolerant to hydroxyurea to receive Ruxolitinib versus standard therapy. Patient-reported symptoms were assessed in this trial using the Myeloproliferative Neoplasm Symptom Assessment Form (MPN-SAF) diary, EORTC QLQ-C30, Pruritus Symptom Impact Scale, and PGIC. Fourteen disease related symptoms were evaluated by the MPN-SAF and resulted as a total symptom score. Ruxolitinib led to significant symptom improvement in the polycythemia patients, as well. [9] A single case in the literature reports on a patient with KIT-mediated systemic mastocytosis associated with myelofibrosis treated with Ruxolitinib. This patient also experienced significant improvement in symptoms and reduction in splenomegaly [12].

Given the remarkable results and quality of life improvement seen in the aforementioned trials when using Ruxolitinib in the treatment of MPNs, Ruxolitinib was trialed in our patient in attempts to alleviate the debilitating constitutional symptoms of systemic mastocytosis. EORTC QLQ-C30, BFI, MPN-SAF, and PGIC were the tools used to assess symptom improvement and improvement in quality of life in our patient [13]. As evident in the QLQ-C30 functional scales graph (Fig. 1a), our patient had improvement in all functional scales and improvement in quality of life and global health status with the use of Ruxolitinib. The QLQ-C30 combined symptoms score (Fig. 1b) decreased dramatically over the 24 weeks on Ruxolitinib, representing the decrease in symptomatology experienced by our patient. The BFI assessment was used on our patient, as fatigue was one of her most debilitating symptoms. Baseline BFI score of 5.8 was decreased to 2.5 on Ruxolitinib, denoting an improvement in fatigue (Fig. 2). The MPN-SAF was used to assess improvement in symptom burden in our patient. Symptoms evaluated by the MPN-SAF assessment include concentration, early satiety, inactivity, night sweats, itching, bone pain, abdominal discomfort, weight loss, and fever. Baseline MPN-SAF total symptom score (TSS) for our patient was near 70 (designated as severe if ≥ 70) and was reduced to 12 by week 24 on Ruxolitinib (Fig. 3). It is noteworthy to mention that higher doses of Ruxolitinib (week 8–12) caused the MPN-SAF TSS to elevate to the 30–40 range, but this was still a significant improvement from baseline. As Ruxolinitib was dose reduced due to worsening anemia in weeks 16–24, the MPN-SAF TSS did decrease proportionately. PGIC was the last assessment tool used to monitor symptoms and improvement in quality of life in our patient. Patient responses range from very much improved to very much worse on this assessment. Our patient’s response for the PGIC was “much improvement” by week 24 on Ruxolitinib therapy.

In addition to monitoring for symptom improvement, adverse events in the setting of Ruxolitinib were evaluated. Weight gain was reported in other trials using Ruxolitinib and was also seen in our patient. No significant adverse events were noted.

Dose escalations were slow and monitored closely since Ruxolitinib was being added to imatinib in our patient. There were no detected toxicities observed in this novel combination of active agents. The combination of imatinib and Ruxolitinib has been published in the literature [14] and no serious toxicity has been demonstrated.

Ruxolitinib is a promising and active agent, especially in terms of symptom improvement and improved quality of life in a variety of patient subsets. Regardless of the underlying disease process, it appears that diseases with proinflammatory and cytokine release mechanisms are obtaining benefit with the addition of Ruxolitinib. In patients’ with systemic mastocytosis with difficult to control symptoms, Ruxolitinib may be a therapeutic consideration. Controlled prospective clinical trials evaluating the role of Ruxolitinib in systemic mastocytosis, post-SM myelofibrosis, and other cytokine-dependent diseases are needed.

### Ethics approval

Not applicable.

### Consent for publication

Consent has been obtained to publish this data and is available to the editors upon request.

### Availability of data and materials

All data is presented in main paper.
